# Exploring factors influencing parents’ adoption intention toward children’s illustrated e-books: A push-pull model perspective

**DOI:** 10.1371/journal.pone.0341651

**Published:** 2026-03-20

**Authors:** Lufei Guan, Wenbo Wei, Mengxi Fu

**Affiliations:** 1 Hunan University of Information Technology, College of Art, Hunan University of Information Technology, Maotang Industrial Park, Xingsha Economic and Technological Development Zone, Changsha, Hunan, China; 2 Graduate School, College of Design, Hanyang University, Seongdong-gu, Seoul, South Korea; 3 Art and Design Department, University of Wales Trinity Saint David, Swansea, United Kingdom; The Chinese University of Hong Kong, HONG KONG

## Abstract

With the development of multimedia technologies, children’s illustrated e-books are getting increasingly popular due to their diverse formats and engaging content. However, few studies have explored the factors influencing parents’ adoption intention toward illustrated e-books for children. This research aims to address this gap by employing the push-pull model to explore the influences of pull factors (i.e., relative advantages, perceived trialability, perceived enjoyment, variety-seeking), push factors (i.e., vision health stress, evaluation cost), and individual characteristics (i.e., age, gender, experience of use) on parents’ adoption intention toward illustrated e-books for children. Data were collected from 348 parents in China and analyzed using structural equation modeling (SEM). The research results suggest that perceived enjoyment and variety-seeking are important pull factors, while evaluation cost serves as a vital push factor. Moreover, children’s gender significantly influences the adoption intention. Parents of boys demonstrated a higher adoption intention than parents of girls. These nuanced insights have practical implications for designers of illustrated e-books for children.

## 1 Introduction

Children’s illustrated e-books, integrating multimedia and interactivity, have emerged as a significant branch of digital publishing, demonstrating potential in enhancing children’s reading interest and comprehension skills [1,2]. However, promoting illustrated e-books in the key context of home reading proves challenging. As primary decision-makers, parents face a dilemma: they are attracted to the interactive nature and reading engagement benefits of digital formats, yet simultaneously worry that screen time may harm children’s visual health. They also have concerns that certain e-book features might negatively impact the quality of parent-child reading sessions and children’s comprehension abilities [3]. This tension between “enhancing engagement” and “managing risks” epitomizes the push-pull dynamics in parental decision-making.

Existing research offers valuable yet fragmented insights into this decision-making process, examining factors such as parents’ own technology usage habits, social influences, and children’s individual characteristics [4,5]. However, a critical research gap is that existing literature lacks an integrated theoretical framework that can simultaneously explain the inherent, competing “push” and “pull” forces within parents’ decision-making processes when adopting new technologies for their children’s leisure reading. Existing models (e.g., UTAUT) effectively explain adoption drivers but fall short in simulating decision processes involving trade-offs and perceived risks- a scenario that is typical for multimedia illustrated e-books for children.

To address this gap, this study introduces the push-pull model as a novel and appropriate analytical perspective. The push-pull framework’s unique applicability lies in its ability to transcend a simple enumeration of influencing factors. Instead, it adopts a systematic theoretical approach, interpreting decision outcomes as the product of interactions between pull factors (attraction factors, such as perceived enjoyment and seeking diversity) and push factors (barrier factors, such as evaluation costs and visual health stress). It poses three research questions:

(1) How do the pull factors influence parents’ adoption intention toward children’s illustrated e-books for children?
(2) How do the push factors influence parents’ adoption intention toward children’s illustrated e-books for children?
(3) How do children’s gender, age, and experience of use influence parents’ adoption intention toward children’s illustrated e-books?

By addressing these questions, this study aims to contribute a balanced and dialectical understanding of parental decision-making. It not only identifies key influencing factors but also clarifies the directionality of these influences (i.e., whether they promote or inhibit adoption), thereby providing a more nuanced theoretical model and actionable insights for the design and marketing of children’s illustrated e-books.

## 2 Theoretical background

In this section, the concept of illustrated e-books and Push-Pull framework were introduced following a discussion about the relationship between related variables.

### 2.1 Children’s illustrated e-books

In the current study, the term “children’s illustrated e-book” refers to a broad category of multimedia reading materials explicitly designed for children, which include electronic books and applications that integrate texts, illustrations, and interactive elements [6]. Specifically speaking, with the development of media information technologies and children’s improved standards for better reading experiences, illustrated books have transcended the boundary of conventional graphic designs and evolved into illustrated e-books involving a wide array of sensory stimuli [5]. Children’s illustrated e-books can be divided into four categories: (a) the electronic version of conventional children’s illustrated books (visual experience); (b) illustrated pop-up books (visual and tactile experiences); (c) audible children’s illustrated books (auditory and visual experiences); (d) multimedia and interactive children’s illustrated e-books (multiple sensory experiences and interactivity). These books share several features. First, they utilize various modes of information communication, such as textual, visual, animated, auditory, and tactile presentations, which can create multifaceted sensory experiences for children [2]. Second, they are usually used with tablet computers, phones, projectors, and similar digital devices. Third, these illustrated e-books offer interactivity between readers and story content, enhancing children’s engagement and allowing them to influence the trajectory of stories [7].

Although the educational potential and limitations of children’s illustrated e-books have been fully discussed in academic literature, existing research remains fragmented in terms of understanding parental decision-making. On one hand, studies has emphasized the benefits of multisensory illustrated e-book in enhancing children’s reading engagement, language skills, and story comprehension [8,9,10]. On the other hand, another body of research has raised significant concerns about potential distractions, disruption of parent-child reading interactions, and the impact of screen time on children’s visual health [2,3].

The popularity of digital reading among young children in China [2] indicates that how to make choices amid these contradictory research findings has become a common and pressing challenge for parents. However, existing studies primarily focus on children’s learning outcomes or the characteristics of books themselves, thus leaving a gap: a lack of systematic investigation into the determinants of parents’ adoption intentions in the specific context of home reading. The family environment presents a unique setting where parents, as primary decision-makers, must weigh known benefits against potential risks. Yet, parents’ decision-making perspective within this distinct context have not been fully explored.

This study aims to address this specific gap. By applying the push-pull model, this study analyzes how two competing forces- pull factors and push factors- jointly influence parents’ intention to use illustrated e-books in children’s home reading. This framework is well-suited to capture decision dynamics in such contexts, as parents’ adoption behavior reflects not merely technological appeal but often involves weighing known risks and barriers. The structure of this model enables a systematic examination of these conflicting factors, thereby offering deeper insights than studies focusing solely on driving factors.

### 2.2 Push-pull framework

The push-pull factors are derived from the paper ‘Laws of Migration’ published by Ravenstein (1885), which are regarded as the foundations of the push-pull model. The push-pull model was originally conceptualised to explain human migration behaviour [11]. Push factors are the forces that drive people away from their place of origin, while pull factors reflect the forces that attract people to their destination [12]. Therefore, push and pull factors represent the two sides of the coin of motivation [12]. The push-pull model is an effective tool for elucidating human switching behaviors that extend beyond migration, which can be applied to analyze consumer behavior when switching brands or products [13]. The push-pull-mooring theory is an extension of the push-pull model, which incorporates push and pull factors to analyze consumer switching behaviors (Moon, 1995). Recent applications of this theory have emerged in the field of social media e-commerce and electronic products [12,13] and in the examination of the decision-making process in leisure travel [13].This framework proves particularly powerful for analyzing trade-off decisions, as it interprets behavioral outcomes as the result of a balance between two opposing forces: attraction and repulsion.

Given the aforementioned research gap, this study adopts the push-pull model as a novel theoretical perspective to systematically explore parents’ adoption intentions toward children’s illustrated e-books in the understudied context of home reading. The adoption intention pertains to parents’ willingness to integrate illustrated e-books into their children’s reading routines. In terms of pull factors, this study identifies attractions from the interactivity and novelty of illustrated e-books, manifested as perceived relative advantages over traditional books, perceived trialability, and perceived pleasurability. Additionally, the pursuit of diversity is incorporated as a key pull factor, responding to parents’ desire for diversified reading experiences for their children.

Conversely, push factors reflect resistance stemming from parental concerns. These include visual health anxieties arising from debates over children’s screen time, as well as assessment costs incurred when diverse formats and content make it difficult for parents to evaluate content appropriateness and quality reliability of illustrated e-books. By applying the push-pull model, this study goes beyond a simple listing of advantages and disadvantages, and provides an integrated perspective to interpret how these competing forces collectively influence parental decision-making within the family environment.

## 3 Development of research hypotheses and models

### 3.1 Pull factors and adoption intention

Relative advantages refer to the extent to which a new thing is perceived as superior to the old one it replaces [14]. Agarwal and Prasad (1997) found that the relative advantages of information and communication technologies (ICT) have significant and positive influences on the perceived ease of use (PEOU), perceived usefulness (PU). And the intention to use ICT products and services. Moreover, an investigation into the switching behaviors of medical consumers revealed that relative advantages have positive influences on consumers’ decisions to switch their preferences. Pi-Jung Hsieh (2021) found that relative advantages can effectively elucidate switching intentions, offering more effective suggestions for personnel in related fields [15]. In the context of this study, with the integration of novel technologies and multimedia elements, children’s illustrated e-books possess explicit and inherent relative advantages over conventional counterparts, which could attract parents to switch behaviors and adopt illustrated e-books for their children. To this end, we hypothesize that:

**H1** Relative advantages will positively affect parents’ adoption intention toward children’s illustrated e-books.

Perceived trialability refers to the extent to which innovations can be tested or experimented with prior to full adoption (Rogers, 1995). Kaye et al. (2019) proposed that the successful introduction of automated vehicles (AV) onto public roadways is dependent on the on-road trial results, which include the public’s acceptance of this technology [16]. Furthermore, Yuen et al. (2020) discovered that perceived trialability has a positive influence on the public’s acceptance of new facilities. A study conducted in Taiwan found that perceived trialability positively influences consumers’ intention to use mobile payment applications for medical services [15]. Within the context of our research, when illustrated e-books for children are introduced to parents and children as innovative reading materials, a trial stage allows both parents and children to experience the functions and services of these e-books. During the trial phase, parents can gain direct perceptions of these illustrated e-books by observing children’s reactions. To this end, we hypothesize that perceived trialability positively affect the adoption intention.

**H2** Perceived trialability will positively affect parents’ adoption intention toward children’s illustrated e-books.

Perceived enjoyment is defined as “the extent to which the activity of using a particular system can be deemed enjoyable, which is irrespective of any performance-related outcomes associated with its use” [17]. Perceived enjoyment focuses on the intrinsic and emotional experiences gained when using a specific product. It depends on the product’s inherent attributes, such as engaging interactions, appealing animations, and pleasant sound effects— these attributes can directly bring immediate pleasure and satisfaction to children (and parents). Therefore, perceived enjoyment emphasizes a deep, immersive experience, motivated by deriving fun from the product being used. Numerous studies have established that perceived enjoyment is an essential driver for adopting technologies or electronic devices. Alzahrani and Mahmud (2017) unveiled that the variables influencing the adoption of online games include perceived behavioral control, subjective norms, attitudes, perceived enjoyment, and flow experience, among which perceived enjoyment shows the most substantial influence [17]. Similarly, Lee (2009) and Lee & Tsai (2010) demonstrated that perceived enjoyment is correlated with attitudes toward products [18]. Besides, perceived enjoyment is found to strongly influence students’ adoption intention toward mobile learning systems [4,18]. Furthermore, the advent of electronic reading has notably improved the reading engagement of children who previously lacked interest in reading. The intuitiveness and interactivity of illustrated e-books stimulate multiple sensory modalities, such as auditory, visual, and tactile experiences, thereby inducing a sense of playfulness and enjoyment, which in turn fosters enthusiasm for reading. Zananda (2020) proposed the potential methods of cultivating children’s enthusiasm for reading [19]. To this end, this study hypothesizes that:

**H3** Perceived enjoyment will positively affect parents’ adoption intention toward children’s illustrated e-books.

Variety-seeking or variety-seeking purchase behavior refers to consumers’ wish to find alternatives even in cases where they derive satisfaction from their current choices [20]. This propensity for brand or product switching can often be influenced by the manipulation of marketing variables (i.e., price, product design, promotion, distribution) or by changes in situational variables. Apart from mere functional considerations, consumers may be driven to explore different products due to intrinsic motivations such as “adding spice to life” or “trying different things” [21]. Adjie et al. (2023) posited that factors like subjective norms, switching costs, and variety-seeking influence users’ switching behaviors from electronic markets to electronic pharmaceuticals [22]. Variety-seeking is from users’ pursuit of external and cognitive benefits derived from switching between different products. It drives not immersion in a single product, but rather the acquisition of novelty and stimulation through experiencing multiple distinct offerings, satisfying psychological needs for exploration and curiosity. For parents, variety-seeking is reflected in their desire to enrich their children’s reading experiences, broaden their knowledge, or avoid monotony by providing illustrated e-books with diverse themes, styles, and interactive forms. Therefore, variety-seeking emphasizes a broad, exploratory experience, motivated by gaining value from changes in product combinations.Therefore, variety-seeking is deemed a pull factor of parents’ intention to adopt children’s illustrated e-books.

**H4** Variety seeking will positively affect parents’ adoption intention toward children’s illustrated e-books.

### 3.2 Push factors and adoption intention

Vision health stress is derived from technostress, which is commonly described as stress experienced when individuals are unable to manage various technological tools and platforms effectively (Maier et al., 2019; Wang et al., 2020). This phenomenon is an unintended consequence of technological advancements and their wide application [23]. Technostress does not arise from a single source. Instead, it emerges from the discordance between user characteristics (such as individual capabilities and values) and environmental factors (such as conditions and demands). In our research, vision health stress is defined as parents’ stress and anxiety concerning their children’s potential vision impairment. As it is usually the parents that choose illustrated e-books for children, parents will consider whether children’s developing eyesight may be too vulnerable to the technostress brought by illustrated e-books. Concerns are particularly amplified regarding issues such as eye fatigue, myopia, and astigmatism. A vision screening of 6-year-old children in 127 Swedish schools demonstrated that 6.6% of these children exhibited a visual acuity (VA) below 0.8 (logMAR 0.1), and 236 of them went through an ophthalmological examination. Among the children who failed to pass the screening, 74.5% showed poor eyesight, quantified as 0.65 (logMAR 0.2) [24]. Moreover, a study conducted during the COVID-19 pandemic showed that a long time of exposure to electronic screens in online courses can increase psychological stress, which can lead to astigmatism. Nowadays, as children’s engagement with multimedia screens continues to rise, parents are concerned about children’s vision health. Meanwhile, Verkijika (2019) discovered that technostress can have a direct negative influence on the intention to continue using digital textbooks [25]. Given the above analysis, our study proposes the following hypothesis:

**H5** Vision health stress will negatively affect parents’ adoption intention toward children’s illustrated e-books.

Evaluation cost refers to the time and cognitive resources expanded in the decision-making process of switching behaviors (Burnham et al., 2003). It is one of the costs that users need to undertake when changing services or products [26]. Within the context of our research, there is a similar cost if users want to switch from conventional illustrated books to electronic counterparts. Parents need to spend time and energy to evaluate whether illustrated e-books are suitable for their children. Despite that the devices for reading these e-books, such as mobile phones and tablet computers, are commonplace in daily life, the diversified forms and content of illustrated e-books underscore the importance of evaluation cost. Different illustrated e-books may vary significantly in their suitability for individual families and children, thereby necessitating a careful evaluation process by users. Given the above analysis, our research proposes the following hypothesis:

**H6** Evaluation cost will negatively affect parents’ adoption intention toward children’s illustrated e-books.

### 3.3 Individual characteristics and adoption intention

In addition to the functions and features of children’s illustrated e-books, parents are likely to consider their children’s unique characteristics when determining the suitability of such e-books to facilitate children’s engagement and knowledge acquisition. These individual characteristics include age, gender, and experiences of using illustrated e-books. The influence of gender on the interaction between individuals and technologies has been a scholarly topic for quite some time, but no conclusion has been made yet [12]. Numerous prior studies on children’s illustrated e-books have proposed that attention and comprehension levels differ across various ages and genders. For example, Ma and Wei (2016) discovered that third-grade children displayed heightened concentration and interest when engaging with illustrated e-books predominantly consisting of pictures, compared to sixth-grade children; boys exhibited better concentration levels when using audible illustrated e-books and multimedia interactive illustrated e-books. Parents’ decision-making in the selection of these e-books can be swayed by children’s interest in these books, which is influenced by their gender and age. Meanwhile, prior experiences of using illustrated e-books could also serve as an influencing factor. Parents are more inclined to adopt illustrated e-books when their children have had experience using them as they believe that experienced children can better adapt to the mediums of e-books. To this end, our research identifies children’s age, gender, and experience of using these e-books as three control variables and studies the correlation between these variables and parents’ adoption intention toward illustrated e-books. It is hypothesized that:

**H7** Children’s age will positively affect parents’ adoption intention toward children’s illustrated e-books.

**H8** Children’s gender will have an impact on parents’ adoption intention toward children’s illustrated e-books.

**H9** Children’s experience of use will positively affect parents’ adoption intention toward children’s illustrated e-books.

**Fig 1:**
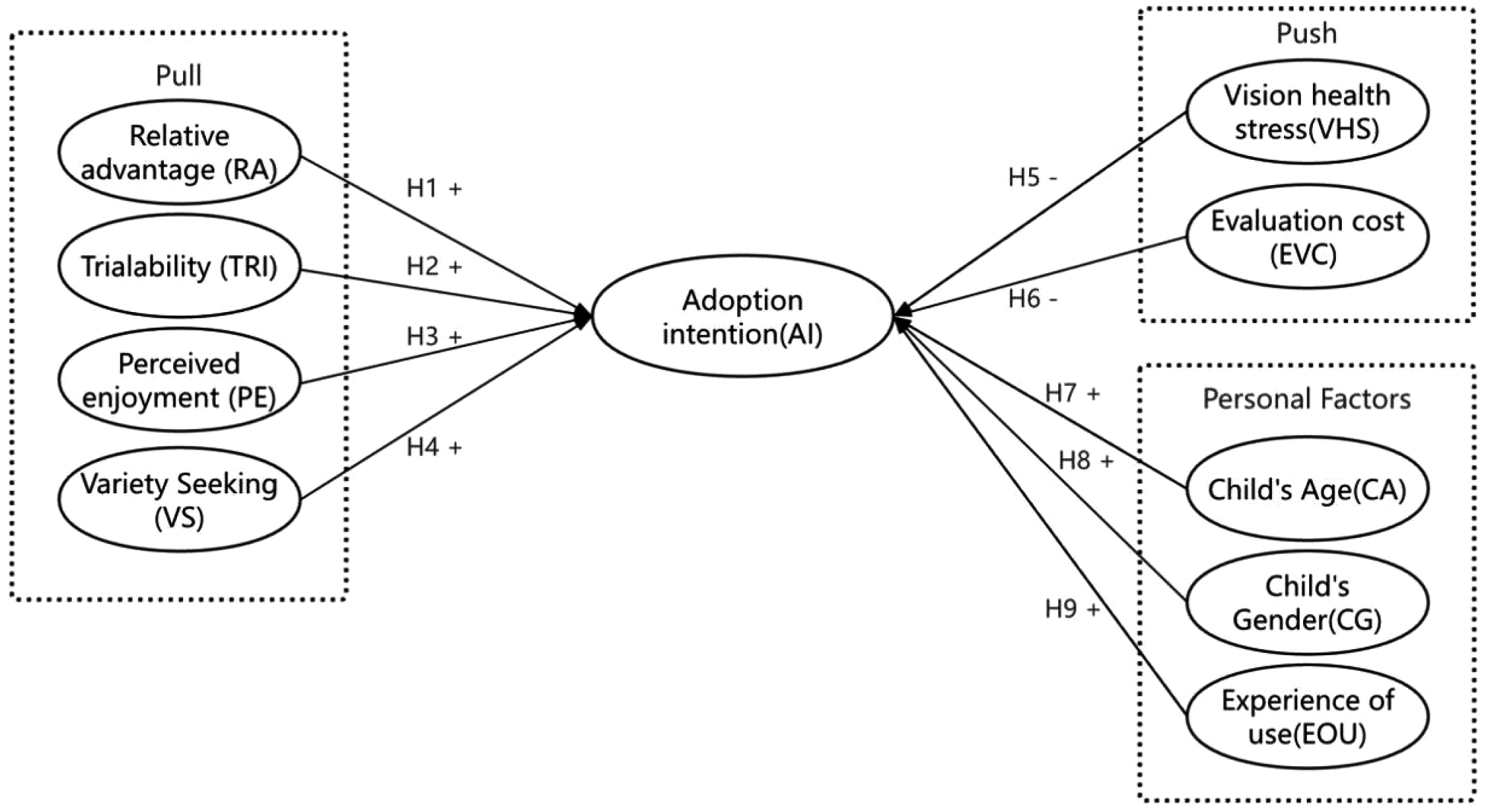
The research model.

This is a research model composed of these assumptions (Fig 1).

## 4 Method

### 4.1 Participants

Since most Chinese families purchase various types of picture books for their children to read, and a significant number of families have experience with multimedia or electronic devices. Therefore,this study used a questionnaire to collect data. An online questionnaire was distributed to parents and parents-to-be in China, and 348 valid questionnaires with an answer time of 30 seconds or more were collected (173 male and 175 female respondents). The recruitment period for this study began on 03/08/2024 and ended on 02/09/2024.Among the children of the respondents, there were 159 boys, 172 girls and 17 unborn children. The age distribution of the children of the participants varied from 109 (31.32%) under the age of 3, 142 (40.8%) between the ages of 3–5, 59 (16.95%) between the ages of 6–8, 31 (8.92%) between the ages of 9–12, and 7 (2.01%) above the age of 12 (Table 1). This study employed non-probability sampling methods to rapidly reach the target population (i.e., Chinese parents). Specifically, we combined a convenience sampling strategy. Questionnaires were primarily distributed online through social media platforms (such as WeChat and Weibo) and parenting online communities. The advantage lies in its ability to collect cost-effective data during the exploratory research phase. However, we also recognize that this method may lead to a sample bias toward parents who frequently use the Internet, possess relatively higher education levels, and reside in urban areas. This could potentially affect the representativeness of the sample for all Chinese parents.

**Table 1:** Demographic information of the participants.

		Number	Percentage
Age	18-30	137	39.37%
	30-40	152	43.68%
	40-50	49	14.08%
	>50	10	2.87%
Gender	Female	173	49.71%
	Male	175	50.29%
Children’s Age	Infertile	16	4.6%
	<3	93	26.72%
	3-5	142	40.8%
	6-8	59	16.95%
	9-12	31	8.92%
	12-15	4	1.15%
	>15	3	0.86%
Children’s Gender	Female	159	45.69%
	Male	172	49.43%
	Unborn	17	4.89%
Total		348	100%

### 4.2 Instruments development

The questionnaire used in this study consisted of two parts. The first part was designed to obtain the demographic information about the participants (i.e., gender, age, children’s gender, and age). The second part consisted of seven subscales with 23 items, which were modified from existing widely accepted measures for the seven latent variables used in this study. All the subscales were in the form of 5-point Likert scale.Written consent was received from all participants.

To be specific, Relative Advantage (RA) and Trialability (TRI) developed based on Pi-Jung Hsieh (2021) [15] “whose original items were developed to evaluate users’ perceptions of the relative advantage of e-Health Pay and their expectations of the trialability of the e-Health Pay application. The adjustments we made were to change “e-Health Pay” to “children’s illustrated e-books” and “cash payments” to “Traditional Picture Books”. Perceived Enjoyment (PE) were revised from Lisana [18] whose original items were developed to evaluate college students’ perceptions of the enjoyment of mobile learning. We used it to assess parents’ and children’s perceptions of the entertaining nature of e-picture books. Variety seeking (VS) were revised from Erasto Akbar Adjie et al (2023) [22] whose original items were developed to test users’ intention to switch from e-marketplaces to e-pharmacies in search of variety. Based on the original title, the question ‘If I find myself liking to buy medicines from an e-pharmacy, I will be reluctant to switch from the app to try something different’ to ’If I find that my child enjoys using illustrated e-books, I don’t use just one illustrated e-book to the exclusion of others. exclusion of others’ to capture whether parents have diverse needs for illustrated e-books.

Vision Health Stress (VHS)was designed referring to the scale developed by Silas Formunyuy Verkijika (2019) [25] whose original project was to assess the effect of Technostress on users’ attitudes towards children’s illustrated e-books and intention to use them. attitudes and intention to use children’s illustrated e-books. Focusing on parents’ anxiety about their children’s visual health, the original items were developed to test the relationship between visual health anxiety and illustrated e-books adoption intention. Evaluation cost (EVC) were revised from Shuang Cheng (2019) [26] whose original items were developed to test the evaluation cost of users switching personal cloud storage service platforms. It was used in this study to assess the evaluation cost of parents’ choice of illustrated e-books.Adoption intention (AI) were modified from the questionnaire developed by Jia Nie et al (2020) [27] whose original items were developed to assess users’ intention to adopt mobile phone English learning check-in, added to the original basis to illustrated e-book background, used to assess parents’ intention to adopt children’s illustrated e-books for their children.

To improve the readability and content validity of the questionnaire, the questionnaire was designed and then initially tested on 20 subjects. Based on the feedback, language expression was adjusted to eliminate possible ambiguity and misunderstanding. We randomized the order of measurement items for independent and dependent variables in the questionnaire. This helps prevent respondents from discerning conceptual relationships between variables, thereby reducing the likelihood of hypothesis guessing and consistency bias. We explicitly stated in the survey instructions that the study was anonymous and data would be used solely for academic purposes, so as to mitigate social desirability bias and encourage honest responses.

### 4.3 Data analysis

Data were analysed by SPSS 26.0,AMOS 23.0 and smart pls3. First, a CFA was conducted to test the reliability and validity of the instrument. Second, Cronbach’s alpha coefficients were calculated to examine the internal consistency of each subscale. These two steps were performed to test the measurement model. Third, HTMT was used to further test discriminant validity.Finally,SEM was conducted to verify the research model.

## 5 Results

### 5.1 The measurement model

The reliability and validity of the measurement model was assessed by the following indicators: Standardized factor loadings, cronbach’s alpha coefficient, convergent validity, discriminant validity and model fit. According to Schumacker and Lomax (2004), standardised factor loadings for items should not be less than 0.50.This criterion was well met by all items in this study. Since the standardized factor loadings for TS4 and BO1 were both higher than 0.70 (ranging from 0.716 to 0.966), these two factors were deleted to improve the appropriateness of the variables（Segars，1997）.Cronbach’s alpha coefficients for all five variables were above 0.80（0.886–0.946）(Table 2),Demonstrates excellent internal consistency.

**Table 2:** Results of construct validity and reliability analysis.

Latent Variable	Measurement Variable	Mean	Std. Dev	Factor Loadings	α	CR	AVE
RA	RA1	2.92	1.19	.796	.881	.898	.688
	RA2			.927			
	RA3			.759			
	RA4			.827			
TRI	TRI1	3.17	1.27	.868	.853	.895	.739
	TRI2			.889			
	TRI3			.821			
PE	PE1	3.3	1.30	.837	.868	.844	.645
	PE2			.833			
	PE3			.734			
VS	VS1	3.14	1.17	.821	.879	.901	.695
	VS2			.887			
	VS3			.823			
	VS4			.802			
VHS	VHS1	3.15	1.29	.941	.864	.853	.664
	VHS2			.670			
	VHS3			.811			
EVC	EVC1	3.01	1.41	.821	.896	.843	.641
	EVC2			.782			
	EVC3			.799			
AI	AI1	3.12	1.30	.880	.868	.872	.695
	AI2			.746			
	AI3			.869			

Convergent validity was evaluated by composite reliability (CR) and average variance extracted (AVE). According to Fornell and Larcker (1981), for all the constructs, CR should not be less than 0.70, and the AVE should be above 0.50. All seven constructs in the present study both satisfy these two criteria (Table 2). As for the discriminant validity of a construct, the square root of AVE must be higher than the correlation coefficients between that and other constructs (Chin, 1998), the measurement model met this standard and suggested a good discriminant validity(Table 3).

**Table 3:** Discriminate validity of the research model.

Constructs	RA	TRI	PE	VS	VHS	EVC	AI
RA	**.829**						
TRI	.478	**.86**					
PE	.511	.471	**.803**				
VS	.543	.438	.441	**.834**			
VHS	−.307	−.278	−.356	−.304	**.815**		
EVC	−.336	−.310	−.334	−.357	.496	**.801**	
AI	.425	.409	.426	.475	−.281	−.333	**.834**

This study set χ^2/^df < 5 as an acceptable level according to the criteria recommended by Hair et al. (2009). Combining the criteria recommended by Hu and Bentler (1999), the results of this study indicated that the fitness of the measurement model is satisfactory with χ^2/^df (chi-square/degree of freedom) = 1.202, AGFI (adjusted goodness-of-fit index) = 0.916, TLI (Tucker-Lewis index) = 0.987, CFI (comparative fit index) = 0.9, RMR (root mean square residual) = 0.062, RMSEA (root mean square error of approximation) = 0.024 (Table 4).

**Table 4:** The goodness of fit indices for the measurement model and research model.

Model	χ^2^	χ^2/^df	AGFI	TLI	CFI	RMR	RMSEA
Measurement model	308.936	1.202	.916	.987	.99	.062	.024
Recommended criteria	p > 0.5	<5.0	>.90	>.90	>.90	<.05	<.08

### 5.2 The structural model

SEM was conducted to verify the research model. Hypothesis testing results show that, except for H6，H8 and H10, the remaining six hypotheses were all supported (Table 5).

**Table 5:** The results hypotheses test.

Hypotheses	Hypotheses path	B	β	S.E.	t	Result
H1	RA → AI	.132	.126	.057	2.311**	Supported
H2	TRI → AI	.200	.172	.067	2.997**	Supported
H3	PE → AI	.235	.230	.059	3.990***	Supported
H4	VS → AI	.334	.305	.064	5.248***	Supported
H5	VHS → AI	−.039	−.035	.062	−.663	Rejected
H6	EC → AI	−.109	−.112	.054	−2.014**	Supported
H7	CA → AI	−.018	−.017	.058	−.318	Rejected
H8	CG → AI	−.285	−.138	.109	2.609**	Supported
H9	EOU → AI	.308	.058	.279	−1.102	Rejected

Relative Advantage (RA) (β = 0.126, P = 0.021)and Trialability (TRI) (β = 0.172, P = 0.003) are positively associated with Adoption intention(AI), supporting hypothesis H1 and H2. Both Perceived Enjoyment (PE) (β = 0.230, P = 0.000) and Variety seeking (VS) (β = 0.305, P = 0.000)have significant positive association with Adoption intention（AI）,supporting hypothesis H3 and H4. Evaluation cost (EC) (β = −0.112, P = 0.044) and Child’s Gender(CG) (β = −0.138, P = 0.009) have negative association with Adoption intention(AI), supporting hypothesis H7 and H9.However, Vision Health Stress（VHS）(β = −0.035, P = 0.527), Child’s Age (CA) (β = −0.017, P = 0.750)，Experience of Use(EOU) (β = 0.058, P = 0.271) have no significant association with Adoption intention(AI), rejecting H5, H7 and H9.In addition, the R-squared value of the dependent variable Adoption intention (AI) was 0.228, indicating that all the independent variables in this study explained 22.8% of the dependent variable AI (Table 6).

**Table 6:** HTMT distinctiveness validity test.

Constructs	AI	EVC	PE	RA	TRI	VHS	VS
AI	–						
EVC	.458	–					
PE	.466	.376	–				
RA	.475	.377	.585	–			
TRI	.355	.355	.546	.548	–		
VHS	.542	.559	.405	.343	.320	–	
VS	.400	.402	.503	.616	.502	.345	–

**Fig 2:**
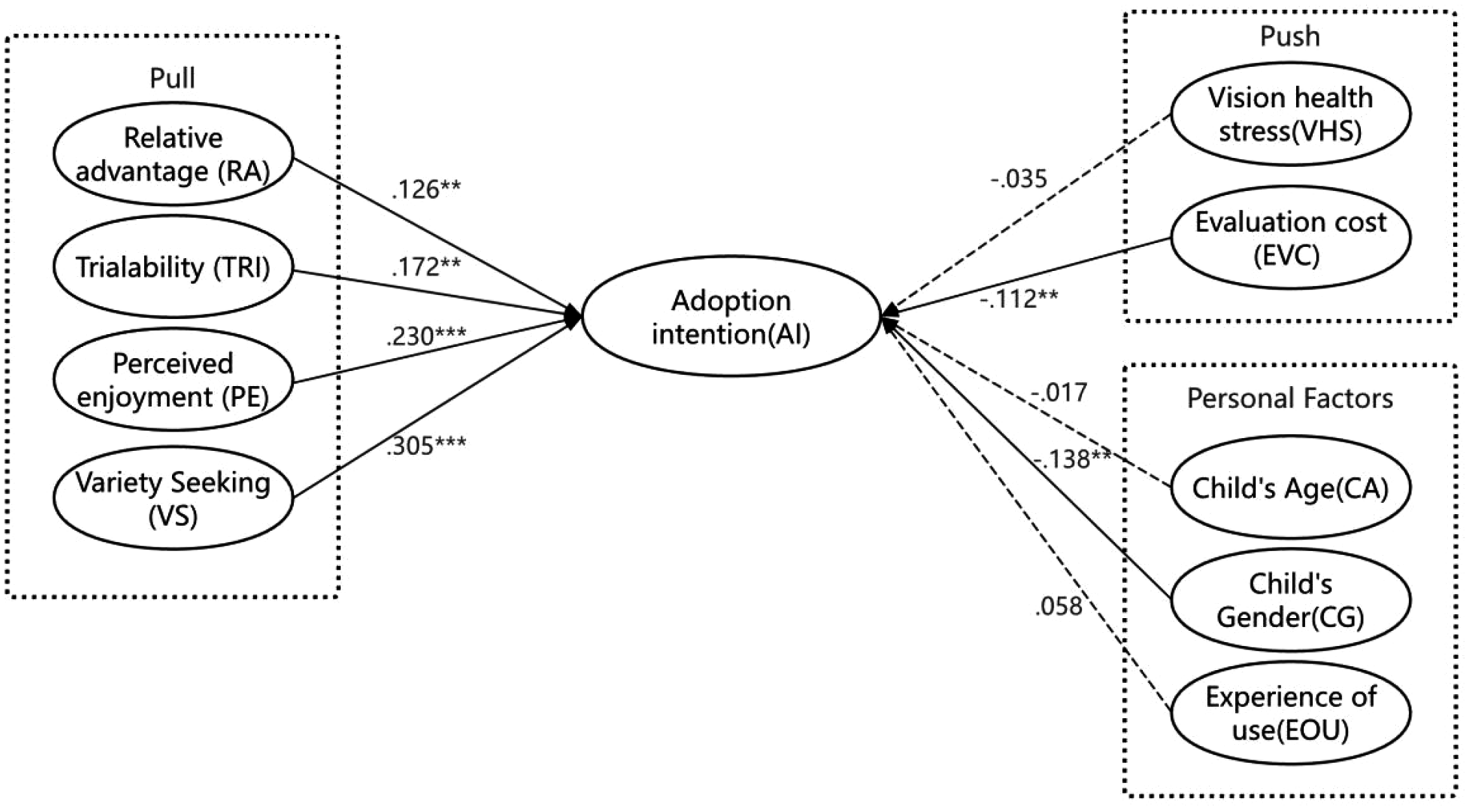
The research model with its standardised coefficients. Dashed lines indicated no significant effects. ***p \.001, **p \.01, *p \.05.

The verified research structural model was shown in Fig 2.

## 6 Discussions

This study initially aimed to address a critical gap in the existing literature: the lack of in-depth understanding of factors influencing parents’ willingness to adopt children’s illustrated e-books in home reading settings, particularly the absence of a theoretical perspective integrating push and pull factors. The findings of this study have successfully addressed this gap. By validating the push-pull model in the Chinese context, we confirm the framework’s effectiveness in explaining parental decision-making and identify perceived enjoyment and variety-seeking as key pull factors, while evaluation cost emerges as the primary push factor. These discoveries offer new insights into understanding parental technology adoption behaviors within the personalized and significant setting of home reading. Here are the detailed research results:

First, in exploring the pull factors influencing parents’ adoption intention (AI) toward children’s illustrated e-books, relative advantages, perceived trialability, perceived enjoyment, and variety-seeking are identified to have significant positive influences on parents’ adoption intention (AI), aligning with previous research results [2,15,19]. In the context of e-book design, designers should emphasize the relative advantages that digital illustrated e-books can offer over traditional printed counterparts, such as enjoyable content, multimedia interactive experiences, and trialability, which cater to the demands for diversification of both parents and children. Among these pull factors, variety-seeking (VS) has the most significant influence on parents’ adoption intention. With a standardized coefficient of 0.305, it is revealed that variety-seeking is a central consideration for parents when selecting children’s illustrated e-books. Perceived enjoyment (PE) is secondary to VS in significance, with a standardized coefficient of 0.230, indicating that the perception of enjoyment derived from using children’s illustrated e-books is the essential experience parents and children expect to gain. To this end, it is suggested that product and service design for children’s illustrated e-books prioritize enhancing entertaining features. For example, sound effects and tactile interactions can be employed to convey the story content, and more interactions can be designed within the story’s plots to maintain entertainment while facilitating comprehension of the content for children.

Moreover, multimedia technologies such as AR can be integrated to create immersive reading experiences for parents and children. The design of children’s illustrated e-books should leverage interactivity and multimedia elements to satisfy parents’ demands for variety-seeking and differentiate their products from conventional illustrated books, thus gaining more favor from parents and children. Relative advantages (RA) and perceived trialability (TRI) have relatively modest influences on the adoption intention with standardized coefficients of 0.126 and 0.172, respectively. The influences of these two variables are closely correlated with the digital characteristics of children’s illustrated e-book platforms and multimedia devices. User perceptions of the relative advantages of illustrated e-books are often informed by their experiential engagement with the products, suggesting that parents are more inclined to choose products with notable strengths and features and greater trialability. To this end, retailers and e-book platforms can enhance the display of relative advantages of their products and improve the services for greater trialability.

Second, in terms of push factors, our research results show that evaluation cost (EC) has a significantly negative influence on parents’ adoption intention, which suggests that evaluation cost is an essential push factor. For parents, the higher the evaluation cost spent while selecting children’s illustrated e-books, the lower their adoption intention. This finding coincides with the research outcomes of Cheng et al. (2019). Consequently, it is essential for suppliers and platforms offering children’s illustrated e-books to mitigate evaluation costs. For example, they can highlight product strengths through comparisons with competing products and provide user guides where the featured functions and content previews are showcased in a clear and intuitive manner, thereby facilitating parents’ understanding of the materials and reducing evaluation costs.

Contrary to initial assumptions, the impact of visual health stress (VHS) on parents’ adoption intention was not statistically significant (β = −0.35). This non-significant finding appears counterintuitive, given that the anxiety over children’s visual impairment has been well documented in existing literature. However, it can be reasonably explained when contextualized within the broader body of literature on parental technology adoption. The educational or developmental benefits of technology often outweigh health-related concerns. Compensatory control theory offers a rational explanation for these findings: parents do not reject technology directly due to health anxieties, but rather engage in risk-benefit trade-offs—choosing to adopt technology while simultaneously planning mitigation strategies (such as strict screen time limits). This compensatory behavior is particularly prominent in Chinese parenting practices, which often emphasize proactive management of children’s environments to foster development. Thus, the non-significant correlation does not imply VHS is irrelevant, but rather indicates its effects are indirect and moderated by perceived value of technology and parents’ subjective sense of agency in managing potential risks through specific interventions.

Third, this study examines the relationships between control variables and parents’ adoption intention. Previous studies have established that children of different ages demonstrate differences in their concentration levels and interest in illustrated e-books. For example, [5] discovered that third-grade children exhibited higher levels of concentration and interest in picture books than sixth-grade children. Nevertheless, our investigation reveals no significant correlations between children’s age (CA) and experience of use (EOU) and parents’ adoption intention toward illustrated e-books. It indicates that for “hedonic-educational” technologies like illustrated e-books, the influence of individual user characteristics may be superseded by the product’s intrinsic attributes. Drawing on the theory of planned behavior, parental decisions may be primarily driven by attitudes toward specific behaviors. In this context, the perceived value is derived from high-quality content and seamless user experience, rather than contextual factors such as the child’s age or prior familiarity. Furthermore, this finding may reflect a unique aspect of parent-child dyads in technology adoption: Parents, as primary decision-makers and purchasers, function as “proxy users”. Their adoption intention is therefore filtered through their own evaluation criteria, which prioritize overall product quality and added value, potentially masking variability introduced by a specific child’s age or prior experience. Thus, the non-significance of CA and EOU does not imply irrelevance, but rather suggests their influence may be indirect and subordinate to the central role of product-centered evaluations in parents’ adoption decisions.

It must be acknowledged that there is a fundamental limitation of our research perspective: the data fully reflect parents’ perceptions and intentions. While parents are the primary decision-makers and purchasers, children are the ultimate users. Their experiences, preferences, and cognitive and emotional responses to the interactive elements, narrative, and visual design of e-books are crucial for comprehensively understanding adoption behaviors and its effects. Our findings, particularly the strong influence of perceived enjoyment, which parents often infer from their children’s reactions, implicitly point to the role of children, but fail to capture it directly. Future research should actively integrate children’s voices through age-appropriate methods to complement the parental perspective outlined herein.

Additionally, our data analysis identifies that there is a significant and negative correlation between children’s gender (CG) and parents’ adoption intention, with a standardized coefficient of −0.138. Notably, parents of boys exhibited a higher adoption intention toward illustrated e-books than parents of girls, as boys at certain ages tend to require increased sensory stimulation to enhance their concentration and comprehension during reading [5]. Consequently, it further indicates that parents are likely to observe children’s responses to illustrated e-books, such as interest and engagement, before deciding on their adoption. This finding may also reflect the potential influence of societal perceptions of gender roles. Traditional notions often characterize boys as more active and requiring external stimulation to sustain attention, a perception well-suited to the multimedia features of illustrated e-books. Consequently, parents may be more inclined to choose such interactive reading formats for boys to support their cognitive and behavioral traits. The gender differences observed in parents’ adoption preferences likely stem not only from observations of children’s individual needs but also reflect deeply ingrained sociocultural gender stereotypes. Future product design and parental education should focus on avoiding the reinforcement of such stereotypes while advocating for more inclusive strategies in selecting reading resources.

## 7 Implications for practice

Our research findings provide empirical evidence for designers of children’s illustrated e-books. This study provides specific guidance for key stakeholders by integrating child-centered design while balancing parental decision-making with children’s experiences as end-users.

For e-book designers, the top priority is enhancing quality through child-centered design. First, designers should focus on creating meaningful interactions that allow children’s choices and actions to directly influence narrative process, as this fosters a sense of autonomy and directly enhances perceptual enjoyment. Second, tto meet the need for variety-seeking, effective strategies involve embedding variability within a single product, such as multiple story endings, open-ended “sandbox” exploration pages, or adjustable difficulty levels in educational games. Finally, learning objectives should be seamlessly added to the story and gameplay rather than being pressented as independent or intrusive quizzes, ensuring education and entertainment complement each other.

For platforms and publishers, the focus should be on enhancing accessibility and supporting parents’ decision-making. A core strategy involves optimizing preview and discovery mechanisms, such as incorporating short video demonstrations and implementing standardized labels for interaction types and learning objectives. This helps users understand products more clearly, significantly reducing evaluation costs. Additionally, platforms should offer refined filtering functions, enabling parents to search based on key factors like educational value, no in-app purchases, and offline accessibility. Simultaneously, promoting trialability through strategies like “first book free” or limited-time full-library trials can effectively leverage perceived trialability to lower adoption barriers.

For educators and parents, this study highlights the importance of informed guidance and selection. Educators can leverage these findings to curate thematic digital reading lists and design supplementary activities linking digital reading with offline games and discussions, thereby enriching learning contexts. For parents, we recommend a three practices: First, engage in active shared reading, deepening understanding and participation by discussing stories and characters’ choices with children. Second, consciously maintain a balanced “media diet” by leveraging the interactive advantages of e-books for reading while preserving traditional print reading as a foundational activity. Third, involve children directly in selecting from vetted book lists, which both respects their rights as end-users and provides parents with valuable insights into children’s preferences.

## 8 Limitations and future work

Our research still has some limitations. First, the research was exclusively focused on the adoption intention of Chinese parents toward children’s illustrated e-books. Consequently, the research results may not be generalizable to other cultural contexts, given the variations in national circumstances and differing levels of multimedia technological advancement across countries. The second limitation of this study lies in the sampling method and sample representativeness. First, while the convenience sampling method we employed effectively collected initial data, it cannot guarantee the statistical representativeness of the sample within the broader population of Chinese parents. Our sample may have failed to adequately cover parents in rural areas, those with lower socioeconomic backgrounds, or those with lower digital literacy. Therefore, the generalizability of the findings should be interpreted with caution. They are more applicable to contexts similar to urban China and parents familiar with the internet. Future research should employ more representative sampling strategies, such as stratified random sampling, to cover a broader range of geographic regions and socioeconomic groups to validate and extend the findings of this study.Third, while vision health stress and evaluation cost are identified as significant push factors influencing parents’ adoption intention toward children’s illustrated e-books, future investigations should explore the potential impact of other variables, such as parental education and digital literacy. Furthermore, this study examined adoption intention solely from the parents’ perspective. While valuable, this approach fails to capture the experiences and preferences of children themselves as end users. Future research should employ child-appropriate methodologies (e.g., observational studies, simplified questionnaires) to directly explore how different e-book features influence children’s enjoyment, comprehension, and motivation for repeated reading. Integrating this child-centered data with parental perspective would yield a more comprehensive model of e-book adoption and effectiveness. Finally, the R^2^ value for adoption intention (AI) in this study was 22.8%, indicating that the independent variables included in the model explain only a limited portion of the variation in adoption intention. This implies that beyond the push-pull factors and individual characteristics examined here, there are other significant variables not incorporated into the model—such as parents’ digital literacy, social norms, and children’s specific interests and preferences. Future research should further explore these potential influencing factors to construct more explanatory theoretical models.

## 9 Conclusion

Based on the push-pull model, this study provides a systematic understanding of the factors influencing parents’ adoption of illustrated e-books for their children. The research conclusively demonstrates that parents’ adoption decisions are primarily driven by perceived intrinsic benefits of digital picture books, namely their relative advantage, trialability, perceived enjoyment, and content diversity, while also being significantly hindered by barriers such as evaluation costs. Crucially, these findings deepen our understanding of technology adoption behavior within family contexts. While health concerns like visual strain exert minimal influence, this likely indicates that parents employ compensatory strategies (e.g., enforcing screen time limits) rather than directly rejecting the technology. A key insight from controlling variables is the significant role of children’s gender, a finding that warrants future research to explore underlying sociocultural or market-driven cognitive factors. Conversely, the non-significance of children’s age and prior experience suggests that parents’ own quality assessments of such products may override children’s specific characteristics.

This study contributes to the literature by validating the applicability of the push-pull framework in the context of hedonic-educational technology adoption within families. However, the generalizability of these findings is limited by the cross-sectional study design and self-reported data. Consequently, future research should adopt longitudinal methodologies to track the evolution of these adoption factors over time. The unexpected role of child gender warrants deeper exploration, alongside investigation into other potential moderators such as parents’ education levels and digital literacy. Another valuable research direction is to investigating the specific nature of compensatory measures adopted by parents to alleviate health concerns.

## Supporting information

S1 Table Raw data. (DOCX)

S1 Appendix Appendix. (DOCX)
