# Supplementary material for: Exploring factors influencing parents’ adoption intention toward children’s illustrated e-books: A push-pull model perspective
Source: PLoS One. 2026 Mar 20;21(3):e0341651. doi: 10.1371/journal.pone.0341651 (PMC13004372; doi:10.1371/journal.pone.0341651)
Supplement: S1 Table — (DOCX) [file pone.0341651.s001.docx]

# Exploring Factors Influencing Parents’ Adoption Intention Toward Children’s Illustrated E-Books: A Push-Pull Model Perspective

My age

| Options | Subtotal | Percentage |
| --- | --- | --- |
| <20 | 5 | 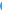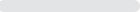1.44% |
| 20-30 | 132 | 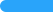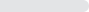37.93% |
| 30-40 | 152 | 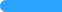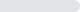43.68% |
| 40-50 | 49 | 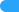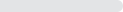14.08% |
| >50 | 10 | 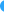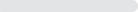2.87% |
| Valid Responses for This Question | 348 |  |

My gender

| Options | Subtotal | Percentage |
| --- | --- | --- |
| Male | 173 | 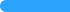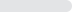49.71% |
| Female | 175 | 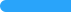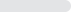50.29% |
| Valid Responses for This Question | 348 |  |

My child's age is：

| 选项 | 小计 | 比例 |
| --- | --- | --- |
| Not yet born | 16 | 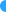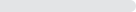4.6% |
| <3 | 93 | 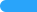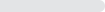26.72% |
| 3-5 | 142 | 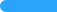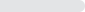40.8% |
| 6-8 | 59 | 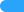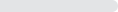16.95% |
| 8-12 | 31 | 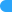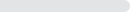8.91% |
| 12-15 | 4 | 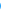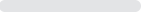1.15% |
| >15 | 3 | 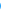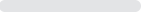0.86% |
| Valid Responses for This Question | 348 |  |

My child's gender：

| Options | Subtotal | Percentage |
| --- | --- | --- |
| Boy | 159 | 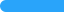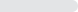45.69% |
| Girl | 172 | 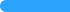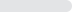49.43% |
| Not yet born | 17 | 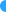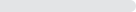4.89% |
| Valid Responses for This Question | 348 |  |

Has my child ever used Illustrated E-Books (broadly defined as any multimedia illustrated storybooks distinct from traditional paper-based picture books, including but not limited to touch-to-read picture books, multimedia animations, interactive picture books, online interactive learning courses, AR/VR story picture books, etc.)?

| Options | Subtotal | Percentage |
| --- | --- | --- |
| A. Yes | 329 | 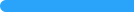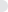94.54% |
| B. No | 19 | 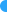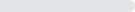5.46% |
| Valid Responses for This Question | 348 |  |

I believe the relative advantages of Illustrated E-Books include…

| Title\Option | Strongly disagree | Disagree | Neutral | Agree | Strongly agree |
| --- | --- | --- | --- | --- | --- |
| Flexible and diverse functions and services | 123(35.34%) | 35(10.06%) | 13(3.74%) | 121(34.77%) | 56(16.09%) |
| Greater ease of use | 71(20.4%) | 78(22.41%) | 60(17.24%) | 79(22.7%) | 60(17.24%) |
| Saving parents time | 80(22.99%) | 77(22.13%) | 41(11.78%) | 100(28.74%) | 50(14.37%) |
| Stimulating children's interest and providing access to more information | 31(8.91%) | 96(27.59%) | 112(32.18%) | 68(19.54%) | 41(11.78%) |

If the Illustrated E-Book is available for trial, I would like to...

| Title\Option | Strongly disagree | Disagree | Neutral | Agree | Strongly agree |
| --- | --- | --- | --- | --- | --- |
| experience the platform and service | 68(19.54%) | 35(10.06%) | 68(19.54%) | 102(29.31%) | 75(21.55%) |
| assess the content's effectiveness and interactive experience | 33(9.48%) | 106(30.46%) | 51(14.66%) | 76(21.84%) | 82(23.56%) |
| test it for a period to see what benefits it brings to my child | 90(25.86%) | 55(15.8%) | 25(7.18%) | 94(27.01%) | 84(24.14%) |

I believe using Illustrated E-Books...

| Title\Option | Strongly disagree | Disagree | Neutral | Agree | Strongly agree |
| --- | --- | --- | --- | --- | --- |
| makes reading and learning more entertaining | 57(16.38%) | 49(14.08%) | 69(19.83%) | 76(21.84%) | 97(27.87%) |
| Children will find it thoroughly exciting | 45(12.93%) | 60(17.24%) | 66(18.97%) | 75(21.55%) | 102(29.31%) |
| parents and children will enjoy using them together. | 84(24.14%) | 43(12.36%) | 37(10.63%) | 75(21.55%) | 109(31.32%) |

I believe using Illustrated E-Books...

| Title\Option | Strongly disagree | Disagree | Neutral | Agree | Strongly agree |
| --- | --- | --- | --- | --- | --- |
| I already possess the relevant environment and equipment | 69(19.83%) | 75(21.55%) | 32(9.2%) | 88(25.29%) | 84(24.14%) |
| It aligns with my current lifestyle | 36(10.34%) | 69(19.83%) | 88(25.29%) | 89(25.57%) | 66(18.97%) |
| It is the lifestyle I favour | 61(17.53%) | 100(28.74%) | 13(3.74%) | 99(28.45%) | 75(21.55%) |

Regarding the diversity of Illustrated E-Books, I believe...

| Title\Option | Strongly disagree | Disagree | Neutral | Agree | Strongly agree |
| --- | --- | --- | --- | --- | --- |
| I prefer purchasing via digital platforms as it's more convenient. | 83(23.85%) | 29(8.33%) | 86(24.71%) | 93(26.72%) | 57(16.38%) |
| If my child enjoys digital picture books, I wouldn't limit myself to just one title while excluding others. | 66(18.97%) | 70(20.11%) | 57(16.38%) | 99(28.45%) | 56(16.09%) |
| Experimenting with different digital picture book products and services doesn't cause me any stress. | 75(21.55%) | 40(11.49%) | 80(22.99%) | 104(29.89%) | 49(14.08%) |
| I wish for my child to experience digital picture books of varied genres and content. | 51(14.66%) | 64(18.39%) | 89(25.57%) | 69(19.83%) | 75(21.55%) |

I believe that in terms of the convenience offered by Illustrated E-Books...

| Title\Option | Strongly disagree | Disagree | Neutral | Agree | Strongly agree |
| --- | --- | --- | --- | --- | --- |
| Children cannot access them at any time | 81(23.28%) | 70(20.11%) | 67(19.25%) | 62(17.82%) | 68(19.54%) |
| Reading and learning activities are restricted to specific locations | 96(27.59%) | 68(19.54%) | 32(9.2%) | 88(25.29%) | 64(18.39%) |
| Requiring one or more electronic devices for use | 50(14.37%) | 113(32.47%) | 24(6.9%) | 74(21.26%) | 87(25%) |
| makes them an inconvenient tool for reading and learning. | 47(13.51%) | 73(20.98%) | 107(30.75%) | 81(23.28%) | 40(11.49%) |

I believe that using Illustrated E-Books...

| Title\Option | Strongly disagree | Disagree | Neutral | Agree | Strongly agree |
| --- | --- | --- | --- | --- | --- |
| can cause eye strain in children. | 74(21.26%) | 48(13.79%) | 72(20.69%) | 115(33.05%) | 39(11.21%) |
| Frequent use places pressure on their visual health. | 88(25.29%) | 59(16.95%) | 9(2.59%) | 79(22.7%) | 113(32.47%) |
| The potential for vision impairment in children causes me anxiety. | 40(11.49%) | 77(22.13%) | 89(25.57%) | 42(12.07%) | 100(28.74%) |

Regarding the evaluation and selection of Illustrated E-Books, I believe I need to...

| Title\Option | Strongly disagree | Disagree | Neutral | Agree | Strongly agree |
| --- | --- | --- | --- | --- | --- |
| devote considerable time and effort to find suitable ones | 91(26.15%) | 60(17.24%) | 50(14.37%) | 75(21.55%) | 72(20.69%) |
| spend some time experiencing digital picture book platforms and services | 83(23.85%) | 72(20.69%) | 28(8.05%) | 66(18.97%) | 99(28.45%) |
| take a considerable period before finally deciding which to use | 90(25.86%) | 70(20.11%) | 16(4.6%) | 83(23.85%) | 89(25.57%) |

Regarding the quality of the Illustrated E-Book application, I believe…

| Title\Option | Strongly disagree | Disagree | Neutral | Agree | Strongly agree |
| --- | --- | --- | --- | --- | --- |
| The loading speed for text and graphics is rather slow | 78(22.41%) | 56(16.09%) | 83(23.85%) | 86(24.71%) | 45(12.93%) |
| The user experience is suboptimal | 68(19.54%) | 78(22.41%) | 68(19.54%) | 57(16.38%) | 77(22.13%) |
| Content and feature information are difficult to navigate | 60(17.24%) | 89(25.57%) | 59(16.95%) | 48(13.79%) | 92(26.44%) |
| Content and interactive elements lack visual appeal | 62(17.82%) | 68(19.54%) | 87(25%) | 64(18.39%) | 67(19.25%) |

Out of habit, I might continue using traditional picture books.

| Title\Option | Strongly disagree | Disagree | Neutral | Agree | Strongly agree |
| --- | --- | --- | --- | --- | --- |
| Change makes me feel pressured and apprehensive. | 68(19.54%) | 70(20.11%) | 78(22.41%) | 40(11.49%) | 92(26.44%) |
| It would be more comfortable and relaxing for me, | 78(22.41%) | 64(18.39%) | 51(14.66%) | 60(17.24%) | 95(27.3%) |
| as I enjoy reading traditional picture books with my child. | 92(26.44%) | 42(12.07%) | 78(22.41%) | 77(22.13%) | 59(16.95%) |

Intention Regarding the Use of Illustrated E-Books

| Title\Option | Strongly disagree | Disagree | Neutral | Agree | Strongly agree |
| --- | --- | --- | --- | --- | --- |
| Illustrated E-Books | 91(26.15%) | 41(11.78%) | 29(8.33%) | 107(30.75%) | 80(22.99%) |
| I intend to use Illustrated E-Books for my child in future. | 48(13.79%) | 64(18.39%) | 99(28.45%) | 66(18.97%) | 71(20.4%) |
| I intend to continue using Illustrated E-Books for my child in future. | 79(22.7%) | 65(18.68%) | 41(11.78%) | 67(19.25%) | 96(27.59%) |

My child uses Illustrated E-Books on average:

| Options | Subtotal | Percentage |
| --- | --- | --- |
| <1 per week | 58 | 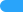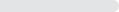16.67% |
| 1-3 per week | 85 | 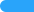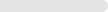24.43% |
| 3-5 per week | 115 | 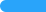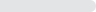33.05% |
| 5-7 per week | 60 | 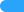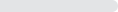17.24% |
| >7 per week | 30 | 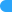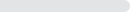8.62% |
| Valid Responses for This Question | 348 |  |

My child's weekly usage time for Illustrated E-Books:

| Options | Subtotal | Percentage |
| --- | --- | --- |
| <1 hour | 102 | 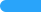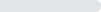29.31% |
| 1-3 hours | 111 | 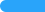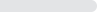31.9% |
| 3-5 hours | 73 | 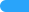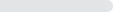20.98% |
| 5-7 hours | 39 | 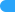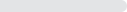11.21% |
| >7 hours | 23 | 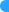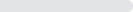6.61% |
| Valid Responses for This Question | 348 |  |
