# Supplementary material for: Exploring factors influencing parents’ adoption intention toward children’s illustrated e-books: A push-pull model perspective
Source: PLoS One. 2026 Mar 20;21(3):e0341651. doi: 10.1371/journal.pone.0341651 (PMC13004372; doi:10.1371/journal.pone.0341651)
Supplement: S1 Appendix — (DOCX) [file pone.0341651.s002.docx]

**Appendix 1**

Comparative Table for Instrument Development

| Original Item from Source | Modifications Made | Final Item in This Study |
| --- | --- | --- |
| The relative advantages of MMPs positively affect a patient’s switching intention. | Replace MMPs with children's illustrated e-books. Change patients' conversion intent to parents' adoption intention. | H1 Relative advantages will positively affect parents’ adoption intention toward children’s illustrated e-books. |
| Trialability is positively related to an individual’s intention to switch to using an MMP app. | Replace MMPs with children's illustrated e-books. | H2 Perceived trialability will positively affect parents’ adoption intention toward children’s illustrated e-books. |
| Perceived enjoyment positively influences students switching intention to adopt ML | Change students switching intention to adopt ML to parents’ adoption intention toward children’s illustrated e-books. | H3 Perceived enjoyment will positively affect parents’ adoption intention toward children’s illustrated e-books. |
| Mooring factors (MRF) influence e-marketplace users’ switching intentions (SI) toward e-pharmacy. | Several attributes have been reported as mooring factors, such as attitudes toward switching, subjective norms, switching costs, prior switching behaviors, and variety seeking.In this study, variety seeking is treated as a driving factor. | H4 Variety seeking will positively affect parents’ adoption intention toward children’s illustrated e-books. |
| Teacher technostress negatively influences intentions to accept and adopt mobile technology. | Change technostress to Vision health stress，Replace adopt mobile technology with parents’ adoption intention toward children’s illustrated e-books. | H5 Vision health stress will negatively affect parents’ adoption intention toward children’s illustrated e-books. |
| Users’ switching cost is negatively related to their intention to switch to the substitute mobile PCSS. | Transition costs comprise: continuation costs, evaluation costs, learning costs, and setup costs.In this study, evaluation costs are treated as a push factor.  Change Switching cost to Evaluation cost，Replace substitute mobile PCSS to parents’ adoption intention toward children’s illustrated e-books. | H6 Evaluation cost will negatively affect parents’ adoption intention toward children’s illustrated e-books. |
| Users’ age has a significant moderating effect on performance expectancy | Replace the mediating effect with the Pull factors. | H7 Children’s age will positively affect parents’ adoption intention toward children’s illustrated e-books. |
| Users’ gender has a significant moderating effect on performance expectancy | Treating gender as an independent variable | H8 Children’s gender will have an impact on parents’ adoption intention toward children’s illustrated e-books. |
| Effort expectation has significant effects on the behavioural intention of people to use digital technology for tackling COVID-19. | Change Effort expectation to experience .Replace use digital technology for tackling COVID-19 to parents’ adoption intention toward children’s illustrated e-books.Replace the mediating effect with the Pull factors. | H9 Children’s experience of use will positively affect parents’ adoption intention toward children’s illustrated e-books. |
